# Isolation and Characterization of Novel Microsatellite Markers for Yellow Perch (*Perca flavescens*)

**DOI:** 10.3390/ijms10010018

**Published:** 2008-12-27

**Authors:** Aibin Zhan, Yao Wang, Bonnie Brown, Han-Ping Wang

**Affiliations:** 1 Aquaculture Genetics and Breeding Laboratory, Ohio State University Aquaculture Research and Development Integration Program, 1864 Shyville Road, Piketon, Ohio 45661, USA. E-Mail: zhan.7@osu.edu; 2 Department of Chemical & Biomolecular Engineering, Ohio State University, 140 West 19th Ave., Columbus, Ohio 43210, USA. E-Mail: wang.934@osu.edu; 3 Ecological Genetics Laboratory, Virginia Commonwealth University, Richmond, Virginia 23284, USA. E-Mail: blbrown@vcu.edu

**Keywords:** Microsatellite, genomic-SSRs, EST-SSRs, yellow perch *Perca flavescens*

## Abstract

To perform whole genome scanning for complex trait analysis, we isolated and characterized a total of 21 novel genomic-SSRs and EST-SSRs for yellow perch (*Perca flavescens*), using the methods of construction of SSR-enrichment libraries and EST database mining of a related species *P. fluviatilis*. Of 16 genomic-SSR primer pairs examined, eight successfully amplified scorable products. The number of alleles at these informative loci varied from 3 – 14 with an average of 8.5 alleles per locus. When tested on wild perch from a population in Pennsylvania, observed and expected heterozygosities ranged from 0.07 – 0.81 and from 0.37 – 0.95, respectively. Of 2,226 EST sequences examined, only 110 (4.93%) contained microsatellites and for those, 13 markers were tested, 12 of which exhibited polymorphism. Compared with genomic-SSRs, EST-SSRs exhibited a lower level of genetic variability with the number of alleles of averaging only 2.6 alleles per locus. Cross-species utility indicated that three of the genomic-SSRs and eight of the EST-SSRs successfully cross-amplified in a related species, the walleye (*Sander vitreus*).

## 1. Introduction

Yellow perch (*Perca flavescens*, Mitchell 1814), widely distributed in fresh waters of the USA and Canada, is an important ecological and aquacultural species, especially in the Great Lakes Region and the Midwestern states of the U.S. This species has been considered one of the most flavorful species among all panfish and carries special advantages in exhibiting a mild taste and firm flesh with low fat and phospholipid content [1]. Due to commercial and recreational overexploitation, harvests have declined since the 1990s. To supply continued high market demand, breeding programs such as O’GIFT (Ohio Genetic Improvement of Farmed-fish Traits) have been launched to improve growth rate and disease resistance of yellow perch. Further investigation of these and other important multigenic traits depends on the availability of molecular genetic markers and use of such markers in efficient breeding programs (e.g. marker assisted selection).

Microsatellite analysis based on polymerase chain reaction (PCR) offers the finest resolution to date for studying molecular variation in perch. Most of the microsatellite DNA markers are Type II markers, which are developed from anonymous genomic sequences. Previously, Type II markers were isolated and characterized to perform the landscape genetic analysis [2] and to evaluate broodstock populations of yellow perch [3]. Comparatively, Type I markers, which are associated with genes of known functions, are more useful for comparative genome mapping [4]. Type I markers often serve as anchorage points for genomic segments. Lack of Type I markers in yellow perch and many other aquaculture species has hindered major progress in genomics and genetic studies in aquatic animals [4]. Although an amount of Type II microsatellite DNA markers were developed for yellow perch, the number of markers is still insufficient for planned QTL analysis of traits such as growth or disease resistance. To increase the numbers of independent simple sequence repeat (SSR) loci available for genomic studies in *Perca flavescens*, we evaluated loci from several microsatellite-enriched libraries and mined online cDNA databases as suggested by a number of researchers [5, 6, 7]. Of particular interest was whether microsatellite markers developed from expressed sequence tag (EST) sequences (i.e. Type I markers) of species related to *P. flavescens* would be sufficiently polymorphic [8].

In the present study, we report bioinformatic mining of the EST database of a related species, European perch (*Perca fluviatilis*), from which we developed polymorphic EST microsatellites for yellow perch. The rates of polymorphism recorded for these markers, both genomic and EST microsatellites, were evaluated by genotyping 30 individuals sampled from a wild population. Additionally, the cross utility of these markers was tested in a related species, the walleye (*Sander vitreus*).

## 2. Experimental Section

### 2.1. EST database mining

To develop EST-SSRs for yellow perch, European perch EST sequences were obtained from GenBank dbEST (http://www.ncbi.nlm.nih.gov/dbEST/index.html). All data were scanned using the software SSR Hunter version 1.3 (http://www.biosoft.net/dna/SSRHunter.htm) using search parameters set to more than seven repetitions for di-nucleotide repeats, five for tri-, four for tetra-, and three for penta-and hexanucleotide repeats.

### 2.2. Microsatellite-enriched library construction

Microsatellite-enriched libraries were conducted using the method described by Li *et al*. [3]. Briefly, the genomic DNA isolated from fin tissue was digested with a restriction enzyme *Sau*3A at 37°C for 3 hours. The fragments with the size range of 0.5 – 2 kb were recovered from an agarose gel. A synthesized adaptor SAUL (A: 5′-GCGGTACCCGGGAAGCTTGG-3′ and B: 5′-GATCCCAAGCTTCCCGGGTACCGC-3′) was ligated to the fragments using T4 DNA ligase. Microsatellite-containing fragments were selectively coupled to biotinylated repeat motifs [(CA)_n_, (GT)_n_, (AAC)_11_, (GAAT)_10_, (ACAT)_11_, (AAAG)_11_, (GTA)_15_, and (AAT)_15_], captured, and washed. Fragments containing microsatellites were ligated to a TOPO vector (Invitrogen) and transformed into competent *Escherichia coli* cells. Positive clones were selected for PCR amplification using M13 universal primers and the PCR products were sequenced.

### 2.3. Sequence analysis and primer design

To exclude duplicates, all sequences, including genomic and EST sequences, were subjected to BioEdit Sequence Alignment Editor Software for grouping clusters using multiple sequence alignment. Microsatellites with the same flanking regions were considered as the same loci. The independent sequences were submitted to the DNA Data Bank of Japan (DDBJ) for homology searches using BLASTN (http://blast.ddbj.nig.ac.jp/top-e.html) against the vertebrate DNA databases to exclude loci previously reported. Sequences with the longest perfect repeats and flanking regions were selected for PCR primer design (Primer Premier version 5.0 software; http://www.PremierBiosoft.com/faq.html). One primer of each primer pair was modified at the 5′-end with an M13 universal tail (5′-CAGTCGGGCGTCATCA-3′) as described by Boutin *et al*. [9].

### 2.4. DNA extraction, PCR amplification and genotyping

A total of 30 adult yellow perch were collected live from a wild population in Lake Wallenpaupack in Pennsylvania, U.S. Individual fin-clips were stored immediately into 95% ethanol. For each specimen, DNA was extracted from 50 mg of tissue according to the methods described by Waters *et al*. [10]. Amplification of microsatellite loci was performed with three primers, the tailed primer, the nontailed primer, and the M13 universal 5′-labelled (FAM, TET, or NED) primer that contained the same sequence as the M13 universal tail. The PCR reaction mix contained approximately 50 ng of genomic DNA, 3 μL of JumpStart RedMix (Sigma), 1.5 pmol of both nontailed and labelled primers, 0.1 pmol of the tailed primer, and 100 μM of spermidine in a total volume of 6 μL. The PCR conditions were programmed as one cycle of denaturation at 95°C for 3 min, followed by 35 cycles of 30s at 95°C, 30s at locus-specific annealing temperature (Table 1), and 45s at 72°C, ending with a final step at 72°C for 5 min. Amplification products were separated using an ABI 3130 Prism DNA genetic analyzer and the genotyping results were analyzed using Genemap® 4.0 software.

### 2.5. Genetic data analysis

For a certain locus, the allele size range (*S*) was directly obtained from the Genemap® 4.0 software. The number of alleles (*A*) and their frequency (*F*), the observed heterozygosity (*H_o_*) and the expected heterozygosity (*H_e_*) were calculated using the computer program POPGENE 32. The Markov chain method [11] was used to estimate the probability of significant deviation from Hardy–Weinberg equilibrium (HWE) and pairwise tests for linkage disequilibrium (LD) were performed using the program GENEPOP online version (http://genepop.curtin.edu.au/) using the default parameters. Significance criteria were adjusted for the number of simultaneous tests using Bonferroni correction [12].

### 2.6. Cross utility

To determine the potential for cross utility, amplification of the identified markers was assessed in one related species, the walleye (*Sander vitreus*). The same PCR conditions and genotyping methods were used as described above except that annealing temperature was re-optimized at each locus.

## 3. Results and Discussion

### 3.1. Genomic-SSRs

A total of 16 sequences derived from the microsatellite-enriched libraries were selected for primer design. The optimization results showed that eight primer pairs could successfully amplify target fragments of the expected sizes. All eight loci exhibited polymorphism in the individuals tested. The numbers of alleles varied from 3 – 14 with an average of 8.5 alleles per locus. The observed and expected heterozygosities ranged from 0.07 to 0.81 and from 0.20 to 0.95, respectively (Table 1). None of the loci showed significant linkage disequilibrium. After sequential Bonferroni correction for multiple tests, five loci were found to depart significantly from Hardy–Weinberg equilibrium (HWE). To exclude the impact of short allele dominance (large allele dropout), data were subject to analysis with Micro-Checker [13]. No evidence for large allele drop-out was found for any of the loci. Further tests indicated that heterozygote deficiency at these loci was responsible for the departure (Table 1). Another possible explanation for the departure from HWE is the dramatic contemporary decline in spawning populations, and consequent non-random mating and genetic bottlenecks [14, 15]. A final possibility is subpopulation structure which cannot be ruled out without further analysis.

### 3.2. EST-SSRs

In the process of EST database mining, a total of 2,226 EST sequences were deposited in GenBank. The mining results showed that 110 (4.93%) sequences contained microsatellites that conformed to our mining criteria (Table 2). As found in other species, di-nucleotide repeats were the most abundant, accounting for 73.64% of all repeats located. This ratio is much higher than has been reported for some other aquatic species such as shrimp, bivalves [5, 6, 16], and other freshwater fish [7]. Surprisingly, the most abundant di-nucleotide repeat type was AG/CT, which is not consistent with reported findings for other fish such as common carp *Cyprinus carpio* [7], pufferfish *Fugu rubripes* [17] and catfish *Ictalurus punctatus* [18] where, in general, the AC/GT repeat type is the most abundant di-nucleotide microsatellite. Similar to our findings, whole genome scanning has indicated that AG/CT is the most abundant type in some aquatic animals such as scallop [19, 20]. Biased sampling, due primarily to the small number of EST sequences examined in this study, may explain the current findings. To further confirm or refine the observation for yellow perch, more sequences or whole genome scanning are needed.

Twenty-three EST-derived sequences were chosen for PCR primer design. Among them, 13 primer pairs (56.5%) amplified products of the expected size. The presence of long introns between primers in genomic DNA, primer sequences spanning across introns and/or mutations, and indels (insertions or deletions) in the primer annealing sites between the two perch species may explain the non-amplification [5]. However, the success ratio we observed for EST-derived microsatellites is slightly higher than those in other studies using the same strategy, such as development of Japanese sea urchin (*Strongylocentrotus intermedius*) using the EST sequences of a related species of purple sea urchin (*S. purpuratus*) [8].

Although we selected relatively long microsatellite regions, the polymorphism assessment results revealed low levels of genetic diversity at these loci. Two or three alleles were detected at most loci and only one locus displayed 5 alleles in the individuals tested (Table 1). Similarly low genetic diversity was also observed in terms of heterozygosity (Table 1). In previous studies where levels of polymorphism have been compared between Type I and Type II microsatellite DNA markers in the same species, the level of polymorphism of Type I markers has usually been observed to be slightly lower [20] but not nearly so dramatic as the differences we observed in polymorphism between EST-SSRs and genomic-SSRs (Table 1). Evolutionary conservation and lower mutation rates within gene-coding sequences is a possible explanation for our observation but does not account for the prior published results. The practical implications are as described by Eujayl *et al*. [21], that a suite of more mutationally-stable EST-SSRs could complement highly variable genomic-SSRs to reconstruct past evolutionary events and to identify regions of genomes that are identical by descent. This would be of particular utility in genomic, gene mapping, and QTL studies across species within *Perca*.

### 3.3. Cross utility

Of eight genomic-SSRs and 12 EST-SSRs, three (37.5%) and eight (66.7%) loci were successfully cross-amplified in the walleye (Table 1), respectively. The cross utility results confirmed that Type I microsatellite markers have higher success ratio than that of Type II microsatellites in the cross-species amplifications among closely-related species. Although the walleye belongs to a different genus, the high cross-amplification ratio was also observed at both genomic-SSR and EST-SSR loci.

## 4. Conclusions

In the present study, a total of 21 novel genomic-SSRs and EST-SSRs for yellow perch (*Perca flavescens*) were developed using the methods of construction of SSR-enrichment libraries and EST database mining of a related species. Compared with the genomic-SSRs, the EST-SSRs for yellow perch displayed a relatively lower level of genetic variability not only in number of alleles but also in heterozygosity. As described in other publications, mining EST databases provides an efficient and low-cost approach to obtaining new microsatellite markers for species of interest. Furthermore, the results also demonstrated the feasibility of microsatellite marker development by EST database mining of a genetically related species in fish.

## Figures and Tables

**Table 1.** Characterization of genomic-SSRs and EST-SSRs for yellow perch (*Perca flavescens*).

Locus name Accession	Primer sequence (5′-3′)	*Ta*	Repeats	*S* (bp)	*A*	*Ho*	*He*	*P*-value	Cross utility (*Ta*; *A*)
YP23† FJ547096	F: M13-TTGGACAAAAATAACTCACT R: AGAGTAGAAATGCGGTTGCT	55	(TTC)_16_	180–210	10	0.8077	0.8620	0.8312	52; 3
YP72† FJ547097	F: AAAGAGAGCAAAGGGGAAGA R: M13-TGTGTAAGAAACAGGCAGGT	55	(GGT)_5_GAA (GGT)_5_GAA(GGT)_16_	255–264	3	0.3846	0.4970	0.4615	54; 2
YP86† FJ547098	F: M13-CCGGCTACTTCATGTTAAAA R: GTGGGAATAAGGGTTAGGCT	55	(AGAT)_14_	331–387	12	0.5185	0.9371	0.0093*	—
YP89† FJ547099	F: ATGGAGATTTACAGCCCCTA R: M13-ACTAATAACCACCATCCTGC	55	(CA)_5_GA(CA)_18_	191–227	6	0.1238	0.6260	0.0000*	—
YP90† FJ547100	F: M13-AGAAAAGAGGGAAAGAAGG R: CCGCTATTTCACTCTGTTTT	52	(GAAA)_16_	123–171	11	0.5556	0.7596	0.8084	—
YP94† FJ547101	F: M13-TTCACATTCAATAGGAGTAGAGT R: CTGTAAAACCATTGCCGATAAA	50	(ACAT)_15_	331–407	9	0.0714	0.8331	0.0003*	—
YP95† FJ547102	F: GTGCCCTTTGTCACCCAT R: M13-GCCCTCATTTATGTCTCTCC	55	(CA)_14_	127–133	3	0.0870	0.3710	0.0001*	52; 1
YP105† FJ547103	F: M13-TAGAAGCAAAACCCGTGA R: TGTCCCTCACCAGCCAGT	55	(CTA)_14_	169–214	14	0.4815	0.9511	0.0028*	—
PFE01# DR730576	F: M13-CTCCCAAAATAAAGCCAATGTC R: ACAGAGTTTCAGGCACTTGTGG	54	(TC)_10_	250–268	2	0.0714	0.0701	0.8907	54; 2
PFE03# DR730639	F: M13-GCAGAAATGCTACATAGATCCT R: AGTCAATATCCTCCAAATGTGC	52	(GT)_16_	124–136	5	0.5714	0.5396	0.8719	50; 3
PFE06# DV671343	F: M13-TTGCCTGAGGTTGTATTGAGAA R: ACAGTCGTAGCAGAGGGTCAC	52	(AG)7	164–176	2	0.0357	0.0357	1.0000	52; 2
PFE07# DV671312	F: M13-CGGCACGAGGGGACTGTAATC R: TGTGCTCTTTCCCTTGTGACCG	50	(AAC)_6_	109–121	3	0.0357	0.1045	0.0018*	54; 1
PFE08# DV671070	F: M13-GTCTTAAACAAGTCTTCATAGCAC R: GGACAGAGAACACATAGAGAATC	56	(TAA)_11_	160–168	2	0.0357	0.0357	1.0000	50; 1
PFE11# DW985750	F: M13-CTTAGACAGACCGACCTACAG R: ATGTCAGCCAAGATGTAATG	50	(TGA)_12_	220–223	2	0.0357	0.0357	1.0000	—
PFE12# DV752650	F: M13-TGCGTGCCAAGGGCGGTGTT R: CCGTCCCCTCAACAAATACC	54	(CCT)_5_	131–149	3	0.0357	0.0708	0.0018*	54; 1
PFE14# DV671188	F: M13-AGCCACAAAGCTGAACATAG R: TGCCATGTTGTATCTCCCAC	52	(AT)_10_	258–264	3	0.1429	0.1351	0.7270	50; 1
PFE15# DR731110	F: M13-GTATTAGTCTATGTATATTGCC R: CGGGATGTCACTTACTTCTC	55	(TATC)_17_	292–296	2	0.0357	0.0357	1.0000	50; 1
PFE19# DV671307	F: M13-TGTCTAACGATTGCTTTTCCT R: CAATGAAAAATAAACATGCGTGACC	56	(AT)_10_	80–82	2	0.0000	0.0701	0.0016*	—
PFE20# DR731052	F: M13-GATCCATCCTGCTCAGACTC R: AAGAGATTGAGTTTGGTAGC	56	(TC)_23_	281–283	2	0.0000	0.0701	0.0016*	—
PFE22# DR730585	F: M13-ATACAGAGGCCTTCATTTGT R: CAGCTACAGTTCATTCTACCT	56	(TA)_9_	280–282	3	0.0714	0.0701	0.8907	—

*T_a_*: annealing temperature (°C); *S*: allele size range (M13 universal tail included);

*A*: number of alleles;

*H_o_*: observed heterozygosity;

*H_e_*: expected heterozygosity;

*P*-value: *P*-values for exact test for Hardy–Weinberg equilibrium (HWE); M13: universal M13 tail (5′-CAGTCGGGCGTCATCA-3′);

Cross utility: primers cross amplified for the walleye (*Sander vitreus*) (*N* = 4);

^#^: EST-SSRs developed for yellow perch;

^†^: genomic-SSRs derived from microsatellite-enriched library;

^*^: departure from HWE after Bonferroni correction.

**Table 2.**
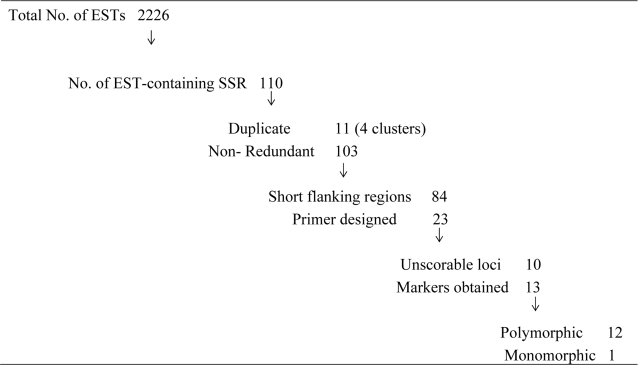
The fates of EST sequences of European perch (*Perca fluviatilis*) used for Type I marker development for yellow perch (*P. flavescens*).
